# Colorectal cancer in south sulawesi: a case-control study for nongenetic risk factors

**DOI:** 10.3389/or.2025.1589655

**Published:** 2025-05-30

**Authors:** Upik A. Miskad, Matthew Martianus Henry, Carissa Ikka Pardamean, Arif Budiarto, Akram Irwan, James W. Baurley, Irawan Yusuf, Bens Pardamean

**Affiliations:** ^1^ Department of Anatomical Pathology, Faculty of Medicine, Hasanuddin University, Makassar, Indonesia; ^2^ Bioinformatics and Data Science Research Center, Bina Nusantara University, Jakarta, Indonesia; ^3^ Faculty of Medicine, Hasanuddin University, Makassar, Indonesia

**Keywords:** colorectal cancer, nongenetic, south sulawesi population, risk factors, lifestyle, socio-demographic

## Abstract

**Background:**

This study analyzed the nongenetic risk factors that contributed to colorectal cancer (CRC) incidence in the South Sulawesi population through a case-control study.

**Methods:**

The sample consisted of 89 cases and 84 controls, aged between 19–86, with 99 males and 74 females from different ethnic groups. Univariate analysis was carried out using chi-square, Fisher’s exact test, t -test, and Mann-Whitney U test. Significant nongenetic risk factors were selected through the logit model L1 regularization, adjusted for age, gender, and ethnicity. The analyzed risk factors were the patient’s weight, height, body mass index (BMI), defecation location, exercise habit, smoking habit, marital status, occupation, education level, and distance to the nearest health center. The estimated odds ratio from the logit model was used to analyze the significance of the selected risk factors.

**Results:**

The significant risk factors from the logit model were smoking habit, education level, marital status, distance to the nearest health center, and weight. CRC cases were more likely to have lower education (OR = 1.819, 95% CI 1.354–2.443), residing in remote areas (OR = 1.44, 95% CI 1.17–1.772), experiencing decreasing weight (OR = 1.03, 95% CI 1.013–1.048). Controls were more likely to be non-smokers (OR = 0.325, 95% CI 0.149–0.707) and unmarried (OR = 0.161, 95% CI 0.036–0.716).

**Conclusion:**

The study determined that other nongenetic risk factors, including education level, distance to the nearest health center, weight, smoking habit, and marital status, contributed to the CRC incidence within the South Sulawesi population. The study emphasized the importance in accounting for these risk factors for further, targeted CRC preventions.

## 1 Introduction

Colorectal cancer (CRC) is a type of epithelial, solid cancer that leads to both mortality and morbidity. Adenocarcinoma is the most common type, accounting for more than 90% of all colorectal carcinoma cases (1). Anatomically, CRC includes both colon and rectal cancers. Its origin remains a subject of debate, centered on the stochastic *versus* cancer stem cells paradigms and hypotheses (2). Considering many resemblances between carcinogenesis and colorectal ontogenesis, remnant fetal stem cells or normal mature colonic cells are thought to possess the potential to develop into cancer stem cells (3, 4). Molecular regulators on gut ontogenesis, such as Nuclear β-catenin, Krüppel-like factor (KLF) proteins, Sonic Hedgehog, or Notch-1 receptor, are observed to be over-expressed in CRC (5, 6, 7, 8). Recent genome-wide association studies (GWAS) have also revealed the role of acetaldehyde as a prognostic risk factor through alcohol consumption habits in light and moderate drinkers (9), highlighting the strong dynamic between environmental risk factors and the molecular mechanisms of onco-pathogenesis.

In 2022, there are 1,926,118 new colorectal cancer (CRC) cases, which places CRC third among other types of cancer in terms of incidence; CRC is also the second most lethal cancer with 903,859 deaths for 2022 (10). Although it is not the most lethal cancer, as of yet, the rates of incidences and mortality are rapidly increasing compared to 2020 (11). Recently, early-onset CRC has also emerged more frequently (12, 13, 14, 15). Some studies locate the tumor of this early-onset cases in the distal (14) and rectal subsites (13). Typically, those with early-onset CRCs are diagnosed at more advanced stages than those with later-onset (16), with dietary and lifestyle changes during the 1950s–1980s hypothesized as the leading cause (17).

Some studies report a regional decline in CRC incidence (12, 13, 14, 15, 18, 19), but this is not the case for many regions in Asia and Africa (20, 21), in which many low- and middle-income countries (LMICs) are situated. Even though high-income countries (HICs) have more CRC incidence (10), the mortality rate is lower compared to LMICs due to healthier lifestyles and public awareness of cancer screening, such as fecal immunochemical test (FIT) and fecal occult blood tests (FOBT) (22). Meanwhile, the inadequate health infrastructure and personnels in LMICs hinders those CRC screening, resulting in failure of incidence and mortality rate reduction (22). Indonesia, to date, remains part of LMICs, and is still struggling with CRC. One study reports that CRC cases in Indonesia are more prevalent in males than females (23). Other studies regarding CRC in Indonesia are divided either geographically (24, 25, 26) or ethnically (27) to accommodate the diverse Indonesian population. The heterogeneous nature requires a comprehensive approach in identifying the unique risk factors for each population.

Guided by the known interplay between environmental and genetic risk factors, this study aimed to connect and decipher the interplay among nongenetic variables linked to CRC occurrence through a case-control study, particularly in the South Sulawesi population. Previously, a GWAS of the same population was conducted (28). Nongenetic risk factors were briefly studied, but limited to the scope of dietary intake variables (29). This study analyzed other nongenetic variables related to patient health, socio-economy, and daily-life routines/behaviors to determine the nongenetic risk factors that were significantly associated with CRC incidence. Through this experiment, this study contributed to the development of targeted CRC prevention for the South Sulawesi population.

## 2 Materials and methods

### 2.1 Patient data collection

This study utilized the same set of observations as in (28, 29). Cases are patients diagnosed with CRC, with the group’s eligibility confirmed by physician examinations. The case samples were collected from seven hospitals: Grestelina Hospital, Wahidin Sudirohusodo Hospital, Akademis Hospital, Hasanuddin University Hospital, Stella Maris Hospital, Hikmah Hospital, and Ibnu Sina Hospital. The seven hospitals are based in Makassar City. Controls are individuals not diagnosed with CRC. This control group was frequently matched to the case group by age, ethnicity, and gender. Through an in-person interview, all patients completed a detailed questionnaire related to demography, family history, smoking behavior, alcohol consumption, and dietary intake summary. Information on clinical and histopathology observations was also collected for the case group. In total, there are 382 questions for the case group and 319 questions for the controls.

Although the samples were collected from hospitals across the city of Makassar, the patients comprised diverse ethnicities in South Sulawesi and across Indonesia. Initially, 162 cases and 193 controls were recruited. However, some patients were unwilling to answer some follow-up questions, which resulted in many missing values for those patients' variables. These observations were not used in this study. The sample used in this study consisted of 173 patients, with 89 cases and 84 controls.

### 2.2 Statistical analysis

Risk factors related to the subject’s health, behavior, and socio-economy were selected from the list of risk factors collected in (27, 28). The risk factors related to the patient’s health were smoking habits, body mass index (BMI), weight, and height. Risk factors related to the participant’s socioeconomic status and daily-life routines/behaviors were distance to the nearest health center, marital status, profession, location of defecation, and education level. The distance to the nearest health center variable contained missing values, which were imputed through linear regression using other variables, with the exclusion of CRC incidence to prevent circularity. While preserving the sample size, this imputation neglects the variance of the missing values and the cause of its randomness, which introduces bias. However, since the percentage of missing samples is relatively small (∼7%), the overall impact of the bias is minimal.

For univariate analysis, nominal risk factors were analyzed using the chi-square test (30). In conditions where the chi-square test assumptions were not met, Fisher’s exact test was utilized (30). Continuous risk factors were analyzed primarily using t-test (31). When the risk factor distribution was ordinal, or the assumptions of the t-test were not met, the Mann-Whitney U test (32) was utilized instead. Multivariate analysis was conducted afterwards using the logit model with L1 regularization (33) to obtain the significant nongenetic risk factors from the non-shrinking logit coefficients. Age, sex, and ethnicity were included in the logit model as the baseline covariate. The L1 regularization coefficient ( C ) candidates ranged between 0 – 1 with inversed regularization strength. A value of C closer to 0 yielded a stricter logit coefficient shrinkage, resulting in fewer risk factors selection. Candidates obtaining the maximum average F1-score from the 10-folds cross-validation was chosen as the best C . All the conducted tests were two-tailed with a p-value level of 0.05.

## 3 Results

### 3.1 Summary of collected samples

The sample collection summary is described in Supplementary Table S1. No different proportion was observed between males and females in either the case or control groups ( p > 0.999). The ages ranged from 19 to 86, with a mean of 53.1 years for the case group and 50.5 years for the control group ( p = 0.12). The proportion of self-reported ethnicity was also similar between the two cohorts, with Bugis and Makassar dominating both groups ( p = 0.86). Other recorded local ethnicities were Mandar, Toraja, Palu, Kolaka, and even ethnic groups from outside of South Sulawesi residing in the city of Makassar.

Assessing from the patient’s health, a mean difference was observed in body weight ( p < 0.001). However, a significant difference was not observed in body height ( p = 0.35). The case group had a higher proportion of smokers ( p < 0.001). Patients in both groups rarely exercised ( p = 0.176). The control group had more access to the nearest health center than the case group ( p < 0.001). Defecation location other than the lavatory significantly increased CRC incidence ( p < 0.001). Marital status also had a significant association with CRC incidence ( p = 0.007), with higher proportion of married patients in the CRC cohort. Regarding education level, both groups reported similar proportions at every level, rendering the risk factor insignificant ( p = 0.493). The same notion occurs in the patient’s occupation in both cohort ( p = 0.51). Univariate analysis revealed that body weight, smoking habit, defecation location, marital status, and distance to the nearest health center significantly contributed to CRC incidence, with the p -value <0.001, except for marital status where p -value = 0.007. Supplementary Table S1 summarizes the obtained p -values for each risk factor. For continuous risk factors analyzed with the Mann-Whitney U test, instead of the mean and standard deviation, median and standard deviation are shown as well.

### 3.2 Significantly-associated nongenetic risk factors

**FIGURE 1:**
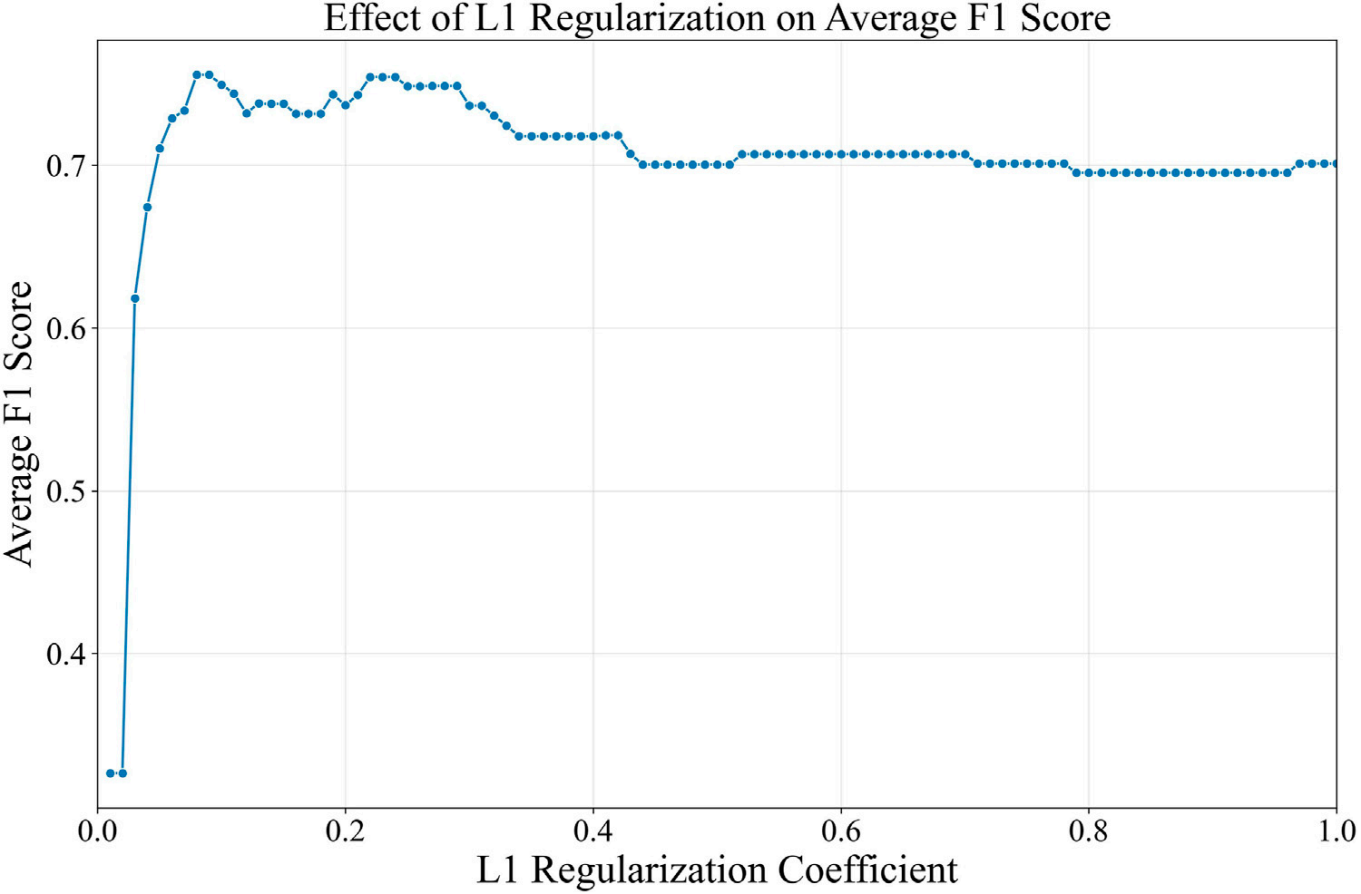
Regularization coefficient and its average F1 score from 10-folds cross-validation.

The risk factors previously described in Supplementary Table S1 were inputted into the L1 logit model to determine significantly-associated nongenetic risk factors to CRC incidence. The nominal risk factors were encoded using one-hot encoding before insertion into the model. Unlike nominal risk factors, the ordinal risk factors have a rank or order that needs to be preserved, in which these risk factors were encoded through label encoding, with higher value representing higher level. The nominal risk factors were gender, ethnicity, defecation location, marital status, smoking habit, and occupation, while the ordinal risk factors were exercise habit and education level. Fisherman occupation caused a quasi-separation leading to their exclusion. From the optimization process, the L1 logit model with C= 0.08 had the highest F1-score of 75.5%. Despite having more risk factors, logit model with C> 0.08 exhibited lower average F1 score (Figure 1). Meanwhile, logit model with C< 0.08 was too sparse, with all risk factors shrunken to 0 in C= 0.01.

**FIGURE 2:**
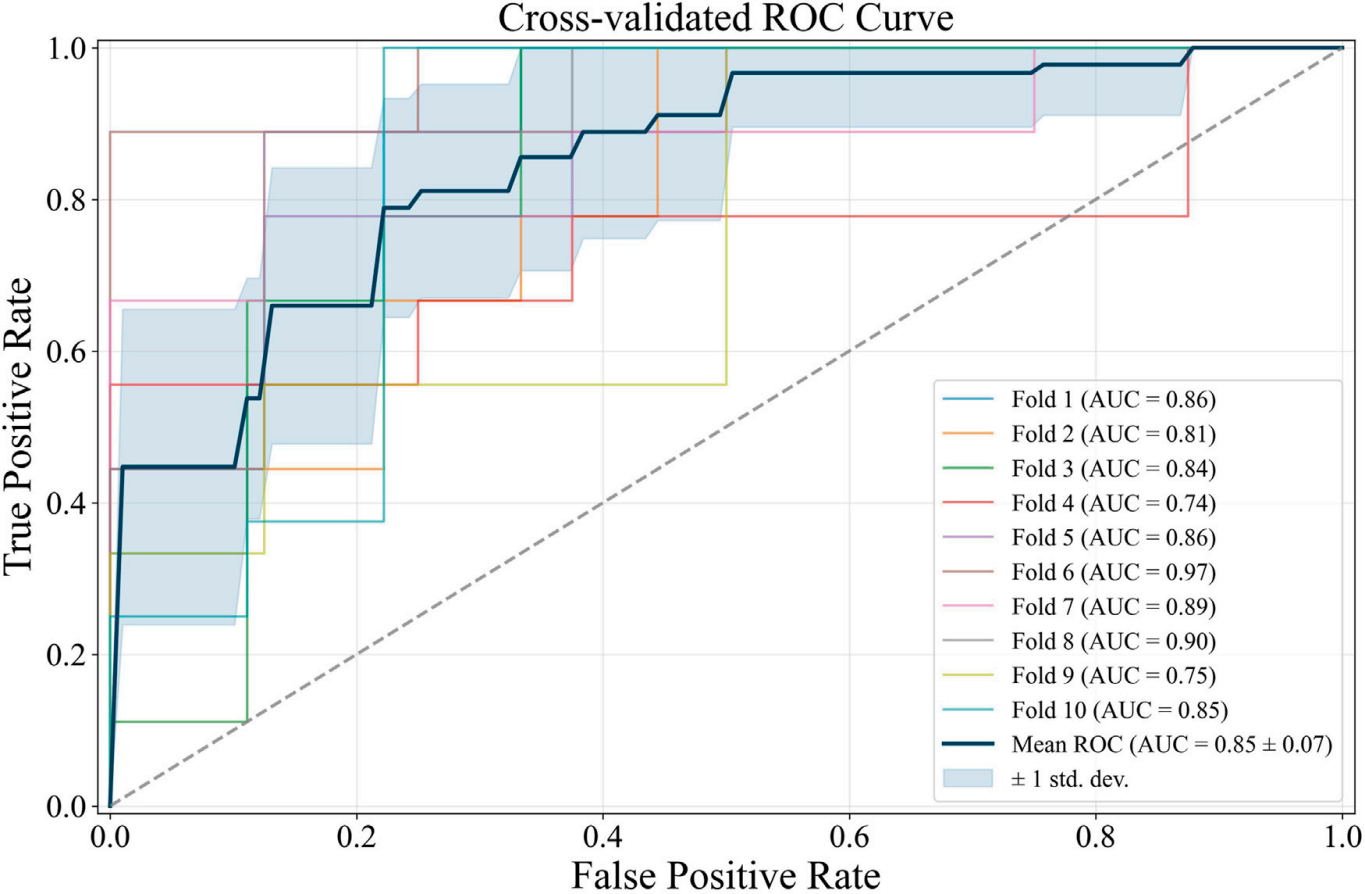
ROC curve of L1 logit with C= 0.08.

Further diagnostics of the L1 logit with C= 0.08 is shown by the Area Under the Curve (AUC) score of each Receiver Operating Characteristic (ROC) curve (Figure 2). Each ROC represents a fold in C= 0.08. The AUC scores for each cross-validation fold vary, with the minimum AUC generated by the fourth cross-validation fold (0.74) and the maximum generated by the sixth cross-validation fold (0.97). As shown in Figure 2, the average AUC score from the 10-fold cross-validation is 0.85, with only a 0.07 standard deviation. This average AUC score exhibits the L1 logit robustness in discriminating the CRC cases and controls in the population, specifically with C= 0.08 and despite the small sample size used. The risk factors from the L1 logit with C= 0.08 were then refitted to unregularized logit (summary in Table 1) to obtain the odds ratios.

**TABLE 1:** Logit summary of the refitted risk factors.

Risk factors	Coefficient	Standard deviation	p -value
Weight	0.030	0.008	<0.001^a^
Smoking Habit			
No	−1.125	0.397	0.005^a^
Marital Status			
Married	−1.830	0.763	0.017^a^
Education Level	0.598	0.151	<0.001^a^
Distance to Nearest Health Center	0.364	0.106	0.001^a^

^a^ indicates significant difference between the case and control groups for that particular variable.

**FIGURE 3:**
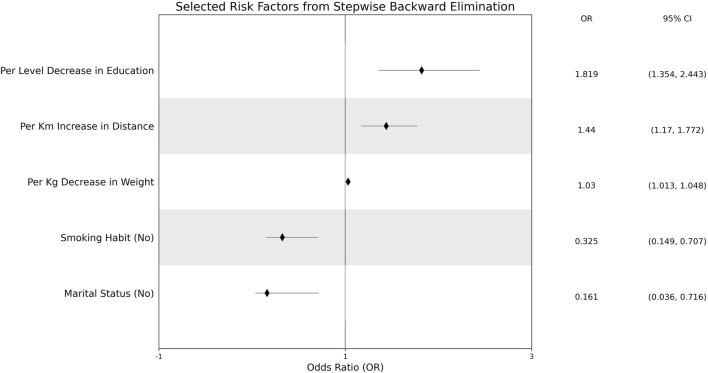
Effect measure plots of significantly-associated risk factors.

The effect measure plot of the odds ratios is shown in Figure 3. A 1-kg increase in a patient’s weight increased the odds ratio of CRC incidence by 3%. A patient with no regular smoking habit had an odds ratio decrease of 67.5%. Patients with lower education levels recorded an 81.9% increase in CRC incidence odds ratio. A married patient had a CRC incidence odds ratio decrease by 83.9%. An increase of 1 km to the nearest health center increased the odds ratio of CRC incidence by 81.9%. None of the adjusting risk factors were associated with CRC incidence in the South Sulawesi population.

## 4 Discussions

In many studies involving diverse populations, smoking habit, either active or passive smoking, is significantly associated with CRC (20, 34, 35). The association is reinforced genetically by the discovery of an associated SNP from the cell adhesion molecule 2 (CADM2) gene, especially in GG and GA allele, that increases CRC risk (36). Based on these results, the significant association between smoking habit and CRC incidence in the South Sulawesi population, in which the absence of smoking habit decreases the chances of CRC (OR = 0.325; 95% CI 0.149–0.707) (Figure 3), and *vice versa*, is predictable and expected. Particularly in this study, the chi-square test on gender and smoking habit found that smoking was more prevalent in males ( p < 0.001), which is proven by the same population distribution in other studies of tobacco use in the South Sulawesi population (37, 38).

Based on Supplementary Table S1, the higher education risk factor alone is independent from CRC incidence ( p = 0.491). However, since education level is treated as an ordinal risk factor in the logit model, a Mann-Whitney U test was also conducted to assess the mean difference in education level in the two cohorts. When applied to the ordinal education level, the Mann-Whitney U test showed a mean difference among the two cohorts, with the education level of the control group averaged in senior high school graduates and the other in junior high school graduates ( p = 0.007). There is no consensus regarding the relationship between education level and CRC incidence. A study in different regions of Indonesia reports socioeconomic factors, including higher education level, to decrease the odds of CRC incidence (39). The increasing odds ratio of education level in this study (OR = 1.819; 95% CI 1.354–2.443) (Figure 3) shows the prevalence of CRC in lower education patients, which aligns with that in other countries (40, 41). Nonetheless, there are other countries that have reported the opposite (42, 43).

Similar to education level, in previous studies, marital status (OR = 0.161; 95% CI 0.036–0.716) shows a diverse association with CRC incidence, with no information on the confounding risk factor if it is associated (41, 44). However, examined from the dietary intake risk factors (29), the confounding factor of marital status in this study is reheated food consumption (Fisher’s exact p = 0.012). A married patient consumes reheated food more often (chi-square = 0.007). In unmarried patients, the condition is not observed (Fisher’s exact p = 0.25) (Table 2). It is speculated that married patients in the city of Makassar, who are older than unmarried patients (Mann-Whitney U p < 0.001), might consume reheated food for faster food serving or other economic factors, such as less frequent food shopping. Nevertheless, due to the sparsity in unmarried group, further study of the dietary intakes in older patients is required.

**TABLE 2:** Contingency table of marital status and reheated foods consumption.

Marital status	Reheated foods consumption	CRC incidence		p -value	Univariate test
		Yes	No		
Married	Seldom	23	57	0.007^a^	chi-square
	Often	63	10		
Unmarried	Seldom	2	9	0.25	Fisher’s exact
	Often	1	0		

^a^ indicates significant difference between the case and control groups for that particular variable.

Patients residing in remote areas near the city of Makassar have limited access to healthcare facilities, especially to ones with comprehensive facilities, which increases the chances of CRC incidence (OR = 1.44; 95% CI 1.17–1.772). The high-quality healthcare facilities in the city of Makassar, based on Google reviews, are densely centralized in Rappocini, Panakkukang, Mariso, Manggala, Ujung Tanah, and Makassar districts (45), which are all located in Southwest Makassar, the central area of Makassar. Therefore, the geographical distribution is imbalanced, with the quality and number of healthcare facilities in Southwest Makassar districts outperforming and outnumbering the healthcare facilities in Tamalanrea and Biring Kanaya, two of the most populated districts in Northeast Makassar, which are also rural districts. A case-control study using other populations has supported the notion (41) that rural area residence contributes significantly to CRC incidence. A study of colon cancer incidence in Yogyakarta, another province of Indonesia, shows no significance of either rural or urban area residence with CRC incidence (26). However, unlike in the city of Makassar, the healthcare facilities in Yogyakarta, especially the hospital networks, are highly comprehensive, even in the rural areas (46). Apart from having easier access to health center facilities, patients residing in urban areas also tend to have more awareness regarding CRC screening and prevention, as proven by studies from other regions (47, 48).

An inverse association was observed between weight and CRC incidence (OR = 1.03; 95% CI 1.013–1.048), which differs from many previously conducted studies (49, 50). However, the inverse is caused by the late measurement of patient weight since it is recorded once the patient has been diagnosed at CRC stage III or IV, as also shown in our previous studies (28, 29).

Based on the identified risk factors, further CRC screening initiatives in Makassar City should prioritize people with low education levels and people living in rural areas, where health access is limited. These two risk factors should also alert the government regarding economic and health infrastructure disparities across the region, which thrive diseases like CRC. Health institutions in the area should strengthen the campaign on smoking dangers and cessation, the danger of often consuming reheated foods, and promoting routine medical checkups in order to facilitate early detection and prevention of CRC.

## 5 Conclusion

As the first to analyze the CRC non-genetic risk factors in the South Sulawesi population, this study delineated the pertinent non-genetic risk factors that could contributed to the CRC incidence in South Sulawesi, especially the city of Makassar. A multivariate logit model was utilized to determine the significant risk factors of CRC incidence for the South Sulawesi population, adjusted by the patient’s age, gender, and ethnicity. Among the inputted health, socioeconomic, and behavioral risk factors, the significantly-associated ones were smoking habit, education level, distance to the nearest health center, marital status, and a patient’s weight. Lower education levels increased the chances of CRC incidence within the population. Unhealthy dietary intake from reheated foods was the influential driver for the significance of the marital status risk factor, with married patients consuming reheated foods more often than the unmarried ones. Similar to other studies that have linked regular smoking with CRC, smoking status was found to be a significant risk factor as well. Additionally, reflecting the state of unequal access to health centers in certain areas surrounding Makassar City, higher chances of CRC incidence was found in patients who inhabit rural areas. Initially, decreased weight was a significant risk factor, but this was due to the late weight and BMI measurements of the patients rather than an independent risk factor. Defecation location also lost its significance in the multivariate analysis. No association was found in this population between CRC incidence and other risk factors, such as exercise habits and a person’s occupation.

## Data Availability

The datasets presented in this article are not readily available because the dataset contains personal information related to the patient privacy. Requests to access the datasets should be directed to bdsrc@binus.edu.
