# Supplementary material for: Colorectal cancer in south sulawesi: a case-control study for nongenetic risk factors
Source: Oncol Rev. 2025 May 30;19:1589655. doi: 10.3389/or.2025.1589655 (PMC12162515; doi:10.3389/or.2025.1589655)
Supplement: Supplementary file 1 [file Table1.DOCX]

Supplementary Material

# Supplementary Table

Table S1. Sample collection summary

| **Risk Factors** | **Case (N = 89)** | **Control (N = 84)** | $\boldsymbol{p}$**-value** | **Univariate Test** |
| --- | --- | --- | --- | --- |
| **Gender** |  |  | >0.99 | chi-square |
| Male | 51 (57.3%) | 48 (57.1%) |  |  |
| Female | 38 (42.7%) | 36 (42.9%) |  |  |
| **Age (years)** | 53.1 ± 13.2 | 50.5 ± 14.5 | 0.12 | t-test |
| **Ethnicity** |  |  | 0.86 | chi-square |
| Bugis | 39 (43.8%) | 45 (53.6%) |  |  |
| Makassar | 24 (27%) | 23 (27.4%) |  |  |
| Other | 26 (29.2%) | 16 (19.0%) |  |  |
| **Weight (kg)** | 54.25 ± 7.9 | 64.08 ± 11.2 | < 0.001* | t-test |
| **Height (cm)** | 160.27 ± 7.81 | 161.32 ± 7.16 | 0.35 | t-test |
| **Smoking Habit** |  |  | < 0.001* | chi-square |
| Yes | 39 (43.82%) | 15 (17.86%) |  |  |
| No | 50 (56.18%) | 69 (82.14%) |  |  |
| **Exercise Habit** |  |  | 0.176 | chi-square |
| Regular | 55 (61.8%) | 61 (72.62%) |  |  |
| Never | 34 (38.2%) | 23 (27.38%) |  |  |
| **Defecation Location** |  |  | < 0.001* | chi-square |
| Lavatory | 71 (79.78%) | 77 (91.67%) |  |  |
| Other | 18 (20.22%) | 7 (8.33%) |  |  |
| **Marital Status** |  |  | 0.007* | chi-square |
| Married | 86 (96.63%) | 70 (83.33%) |  |  |
| Unmarried | 3 (3.37%) | 14 (16.67%) |  |  |
| **Occupation** |  |  | 0.51 | Fisher’s exact |
| Farmer | 18 (20.22%) | 13 (15.48%) |  |  |
| Fisherman | 3 (3.38%) | 0 (0%) |  |  |
| Laborer | 15 (16.85%) | 7 (8.33%) |  |  |
| Civil Servant | 15 (16.85%) | 17 (20.24%) |  |  |
| Private Employee | 10 (11.24%) | 7 (8.33%) |  |  |
| Freelance | 10 (11.24%) | 7 (8.33%) |  |  |
| Other | 18 (20.22%) | 33 (39.29%) |  |  |
| **Education Level** |  |  | 0.493 | Fisher’s exact |
| Elementary School | 10 (11.24%) | 7 (8.33%) |  |  |
| Junior High School | 12 (13.48%) | 4 (4.76%) |  |  |
| Senior High School | 37 (41.57%) | 27 (32.14%) |  |  |
| Bachelor | 21 (23.6%) | 38 (45.24%) |  |  |
| Graduate | 2 (2.24%) | 2 (2.39%) |  |  |
| Doctorate | 0 (0%) | 3 (3.57%) |  |  |
| Other | 7 (7.87%) | 3 (3.57%) |  |  |
| **Distance to Nearest Health Center (km)** | 2.0 ± 3.17 | 1.11 ± 1.46 | < 0.001* | Mann-Whitney U |

## * indicates significant difference between the case and control groups for that particular variable
